# Ponatinib alleviates non-alcoholic steatohepatitis through TFEB-mediated autophagy

**DOI:** 10.3389/fphar.2024.1505768

**Published:** 2025-01-07

**Authors:** Zhuomiao Lin, Meiqing Yang, Xihui Yu, Guozhu Tan, Jiahong Zhong

**Affiliations:** ^1^ Department of Clinical Pharmacy, Meizhou People’s Hospital (Huangtang Hospital), Meizhou, China; ^2^ Joint Shantou International Eye Center, Shantou University and The Chinese University of Hong Kong, Shantou, China; ^3^ Department of Pharmacy, The Second Affiliated Hospital of Shantou University Medical College, Shantou, China; ^4^ Department of Orthopaedics and Traumatology, The Seventh Affiliated Hospital, Southern Medical University, Foshan, China

**Keywords:** ponatinib, NASH, autophagy, TFEB, methionine and choline deficient diet

## Abstract

**Objective:**

Non-alcoholic steatohepatitis (NASH) is a progressive liver disease with lipid accumulation, inflammation, and liver fibrosis. Ponatinib, a third-generation tyrosine kinase inhibitors for the treatment of chronic myeloid leukemia, was found to improve metabolic disorders in mice. However, the role of ponatinib in liver inflammation and fibrosis remains to be elucidated. Here we aimed to determine the effect of ponatinib in non-alcoholic steatohepatitis.

**Methods:**

We explored the function and mechanism of ponatinib using a mouse model of NASH induced by a methionine and choline deficient (MCD) diet and LO2 cells cultured in MCD mimic medium.

**Results:**

Here, we found that ponatinib reduced liver lipid deposition, fibrosis, and inflammation induced by MCD diet without affecting body weight and blood glucose. Meanwhile, we found that ponatinib attenuated steatohepatitis and inflammation in LO2 cells induced by MCD mimic medium. We further discovered that the expression levels of LC3II and lysosomal associated membrane protein 1 (LAMP1) were reduced and the expression level of p62 was upregulated in both mouse and cell models, suggesting that autophagy was inhibited, which was restored by ponatinib treatment. In addition, transcription factor EB (TFEB) is a major regulator of autophagy and lysosome biogenesis and the transcription and protein expression levels of TFEB were decreased in steatosis hepatocytes, which could be ameliorated by ponatinib treatment.

**Conclusion:**

These results revealed that the beneficial effects of ponatinib on NASH via TFEB-mediated autophagy.

## Introduction

NASH, a progressive form of non-alcoholic fatty liver disease (NAFLD), constitutes a spectrum of liver disorders that stem from benign fatty liver disease (simple steatosis) and may progress to NASH, cirrhosis, and even hepatocellular carcinoma and it is associated with an elevated risk of cardiovascular events, heart failure, and diabetes (Powell et al., 2021; Pipitone et al., 2023). The current clinical strategy for the treatment of NASH is to use insulin sensitizers, hypolipidemic drugs and anti-inflammatory drugs to improve the metabolic syndrome and improve liver function, which lacks the specific treatment of NASH (Targher et al., 2021). Consequently, there is an urgent need to identify drugs that are more specific, highly effective, and possess fewer side effects for the treatment of NASH.

The predominant pathological manifestation of NASH is the substantial accumulation of lipid droplets (LDs) within hepatocytes. It is currently believed that the formation of LD in the liver is an adaptive response to the increase in free fatty acid (FFA) and the synthesis of new lipids (Scorletti and Carr, 2022; Zhang et al., 2018). In pathological conditions, on the one hand, the imbalance of LD synthesis and degradation induces excessive deposition of intracellular lipids and results in inflammation and fibrosis in hepatocytes; on the other hand, the intermediate products of lipid metabolism bring about cell dysfunction (Zhang et al., 2018; Scorletti and Carr, 2022). Therefore, the key to controlling the NASH is to regulate the storage of LD (Scorletti and Carr, 2022; Zhang et al., 2018).

Autophagy is an important physiological process mediated by intracellular lysosomes for the removal of pathogens, damaged organelles, and misfolded proteins to maintain cellular homeostasis (Ren et al., 2024). In recent years, a series of studies have shown that hepatic autophagy is involved in the regulation of LD, and the reduction of autophagy will lead to the progression from NAFLD to NASH (Ren et al., 2024; Filali-Mouncef et al., 2022). In addition, studies have shown that drugs with hepatic steatosis side effects are associated with their inhibition of hepatic autophagy (Stankov et al., 2012). These findings support the important role of autophagy in the maintenance of hepatic fat levels and the abnormal autophagy plays an important role in the pathogenesis of NAFLD, suggesting that autophagy may be an innovative strategy for the pharmacological treatment of NAFLD (Yan et al., 2022).

Ponatinib is a multi-targeted tyrosine kinase inhibitor for the treatment of chronic myeloid leukemia and Philadelphia chromosome-positive acute lymphoblastic leukemia (Attwa et al., 2024). Previous studies have shown that ponatinib plays a therapeutic role in the intervention of non-cancer diseases (Zhang et al., 2023; Kang et al., 2016; Qu et al., 2015; Choi et al., 2018). Recent animal studies have revealed that treatment with ponatinib led to improvements in the metabolic profiles of apolipoprotein E-deficient mice and ob/ob mice, suggesting a potential key role for ponatinib in the regulation of lipid metabolism (Pouwer et al., 2018; Lin et al., 2022). However, the potential benefits of ponatinib in the context of NASH, particularly regarding its effects on liver inflammation and fibrosis beyond hepatic steatosis, remain poorly understood. Therefore, we investigated the impact of ponatinib on NASH in this study.

## Materials and methods

### Reagents

Ponatinib was obtained from Selleck (Houston, TX, United States). Fetal bovine serum (FBS) and culture medium were purchased from Gibco (Carlsbad, CA, United States). Palmitic acid, Oleic acid and Oil Red-O were obtained from Sigma-Aldrich (St. Louis, MO, United States). The MCD diet and methionine and choline sufficient (MCS) diet were purchased from Research Diets Inc (Bergenfield, NJ, United States). Tissue-Tek OCT compound was bought from Sakura (Tokyo, Japan). Antibodies against p62, LC3B, LAMP1, TFEB, GAPDH were purchased from Cell Signaling Technology (Danvers, MA, United States).

### Animal study

Six-week-old male wild-type mice with a C57BL/6J genetic background were purchased from Zhuhai BesTest Bio-Tech Co., Ltd. (Zhuhai, China). Before the formal experiments, the mice were made to adapt to feeding for 2 weeks and were housed in a standard feeding environment with a humidity of 50%–70%, a temperature of 23°C–25°C, and a 12–12 h light/dark cycle with lights, where they could freely eat and drink water. All of the animal experiments were carried out in line with the NIH Guide for the Care and Use of Laboratory Animals (revised in 1996), and were approved by the Laboratory Animal Ethics Committee of Meizhou People’s Hospital (Meizhou, China). The experimental design adhered to the 3R principles (replacement, reduction, and refinement) to minimize animal discomfort and reduce the number of mice used. After drug administration, mice were euthanized by inhalation of a dose of 5% isoflurane. During the preliminary study, a sample size calculation was conducted to determine the minimum number of mice necessary to detect a statistically significant difference in steatosis between MCD treated mice and (MCD + ponatinib) -treated mice. Using a two-tailed test, a sample size of five mice per group was determined to be necessary to detect a difference with 95% confidence and 80% power. After a 2-week adaptation, the mice were randomly divided into four groups, with five animals in each group. A NASH mouse model was established in eight-week-old male C57BL/6 mice by administering a MCD diet for 4 weeks. Concurrently, a control group was established by feeding eight-week-old male mice a MCS diet for the same duration. The drug intervention groups (MCD + ponatinib and MCS + ponatinib) were treated with 5 mg/kg ponatinib (dissolved in 25 mM citrate buffer, pH 2.75) by intragastric administration for 4 weeks, while the control group received vehicle (citrate buffer, pH 2.75). Body weight was measured weekly. At the end of the experiment, mice were euthanized after an overnight fast, and the blood and liver tissue samples were collected for subsequent analysis.

### Blood glucose detection

The blood glucose levels of mice were measured using a glucose meter as previously described (Melander et al., 2024). The mice were fasted for 12 h before performing the glucose tolerance test (GTT). Mice were injected intraperitoneally with 1.5 g/kg glucose for the detection of GTT. Blood glucose was measured at 30 min, 60 min, 90 min and 120 min after injection, respectively. The plasma insulin levels of mice were detected using an insulin ELISA Kit (RayBiotech, Norcross, GA, United States) according to the manufacturer’s instructions. The homeostasis model assessment of insulin resistance (HOMA-IR) index was calculated using the formula: [fasting blood glucose (mmol/L) × fasting insulin (mIU/L)]/22.5.

### Histological analysis

Histological analysis was performed as previously described (Song et al., 2023). Liver tissues were fixed in 4% paraformaldehyde, dehydrated, and embedded in either Tissue-Tek OCT compound or paraffin. Paraffin-embedded liver sections were performed for Hematoxylin and Eosin (H&E) staining to assess steatosis and inflammation. Frozen liver sections were stained with Oil Red O to observe lipid accumulation in the liver. All images were captured using an optical microscope (ZEN; Carl Zeiss AG, Oberkochen, Germany). The area of hepatic Oil Red O staining was quantified using ImageJ software. The NAFLD Activity Score (NAS) is a semi-quantitative scoring system used to assess changes in NAFLD/NASH during treatment trials as previously described (Song et al., 2023). It was calculated by summing the scores for steatosis, lobular inflammation, and hepatocellular ballooning. As previously mentioned, the determination of liver pathology is based on the NAFLD activity score and the NAFLD fibrosis score. The pathological feature scores for steatosis (0–3), lobular inflammation (0–3) and hepatocellular ballooning (0–2) were diagnosed by experienced pathologists. Generally, a NAS score of 5 or higher indicates a diagnosis of NASH.

### Plasma measurements

The blood samples collected in EDTA tubes were centrifuged at 1,000×g for 20 min in 4°C, and the supernatant was collected. The plasma concentration of alanine aminotransferase (ALT), aspartate aminotransferase (AST), triglycerides (TG), total cholesterol (TC), high-density lipoprotein cholesterol (HDL-C) and low-density lipoprotein cholesterol (LDL-C) was measured using an automated analyzer (Mindray, Shenzhen, China).

### Hepatic lipid analysis

Liver homogenate was prepared at a 2% concentration in PBS using a tissue homogenizer. The content of liver triglycerides, cholesterol and FFA was quantified using the triglyceride, cholesterol and FFA assay kits (Applygen Technologies, Beijing, China), following the manufacturer’s instructions.

### RNA extraction and quantitative reverse transcription polymerase chain reaction (RT-qPCR)

Total RNA was extracted from liver tissue using Trizol reagent. The RNA (1 μg) was reverse transcribed into cDNA using the QuantiTect Reverse Transcription Kit (Qiagen, Hilden, Germany). Real-time PCR was carried out using the SYBR Green PCR Master Mix (Invitrogen, Carlsbad, CA) on LightCycler 480 (Roche, Basel, Swiss). β-actin was used as an internal control, and the fold change in gene expression was calculated using the 2^−ΔΔCT^ method. The primers used in the RT‒qPCR assay were listed in Table 1.

**TABLE 1:** Sequences of primers.

Primer description		Primer sequence
humIL1b	Foward	CCA​CAG​ACC​TTC​CAG​GAG​AAT​G
	Reverse	GTG​CAG​TTC​AGT​GAT​CGT​ACA​GG
humIL6	Foward	AGA​CAG​CCA​CTC​ACC​TCT​TCA​G
	Reverse	TTC​TGC​CAG​TGC​CTC​TTT​GCT​G
humTnfa	Foward	CTC​TTC​TGC​CTG​CTG​CAC​TTT​G
	Reverse	ATG​GGC​TAC​AGG​CTT​GTC​ACT​C
humTFEB	Foward	CCT​GGA​GAT​GAC​CAA​CAA​GCA​G
	Reverse	TAG​GCA​GCT​CCT​GCT​TCA​CCA​C
humTFE3	Foward	GAT​CAT​CAG​CCT​GGA​GTC​CAG​T
	Reverse	AGC​AGA​TTC​CCT​GAC​ACA​GGC​A
humMITF	Foward	GGC​TTG​ATG​GAT​CCT​GCT​TTG​C
	Reverse	GAAGGTTGGCTGGACAGGAGTI
humCTSF	Foward	ACA​GAG​GAG​GAG​TTC​CGC​ACT​A
	Reverse	GCT​TGC​TTC​ATC​TTG​TTG​CCA
humDPP7	Foward	GAT​TCG​GAG​GAA​CCT​GAG​TG
	Reverse	CGG​AAG​CAG​GAT​CTT​CTG​G
humTPP1	Foward	GAT​CCC​AGC​TCT​CCT​CAA​TAC
	Reverse	GCC​ATT​TTT​GCA​CCG​TGT​G
humGAPDH	Foward	GTC​TCC​TCT​GAC​TTC​AAC​AGC​G
	Reverse	ACC​ACC​CTG​TTG​CTG​TAG​CCA​A
musIL1b	Foward	GCC​ACC​TTT​TGA​CAG​TGA​TGA​G
	Reverse	TGA​TAC​TGC​CTG​CCT​GAA​GC
musIL6	Foward	TAC​CAC​TTC​ACA​AGT​CGG​AGG​C
	Reverse	CTG​CAA​GTG​CAT​CAT​CGT​TGT​TC
musTnfa	Foward	AGG​CAC​TCC​CCC​AAA​AGA​TG
	Reverse	CCA​CTT​GGT​GGT​TTG​TGA​GTG
musTFEB	Foward	GCG​AGA​GCT​AAC​AGA​TGC​TGA
	Reverse	CCG​GTC​ATT​GAT​GTT​GAA​CC
musTFE3	Foward	CTA​TCT​TCC​AGG​AGG​CAC​TGC​A
	Reverse	CTC​GCG​TTT​GAT​GTT​AGG​CAG​C
musMITF	Foward	GAT​CGA​CCT​CTA​CAG​CAA​CCA​G
	Reverse	GCT​CTT​GCT​TCA​GAC​TCT​GTG​G
musCTSF	Foward	CAC​AGC​TCA​GTA​TGG​GAT​CAC​C
	Reverse	TTG​GCT​GGA​CTC​ATC​TTC​CTG​C
musDPP7	Foward	CCA​AAG​GAC​CTG​ACT​CAG​CTC​T
	Reverse	GCC​CTC​ATT​CAA​CAG​CCG​TTG​A
musTPP1	Foward	CAC​TGT​GTC​TGG​CTC​ATT​GCT​G
	Reverse	TGA​CAG​AAG​GCT​AAC​GCT​GGC​A
musGAPDH	Foward	GAA​GGG​CTC​ATG​ACC​ACA​GT
	Reverse	GGA​TGC​AGG​GAT​GAT​GTT​CT

### Western blot analysis

Western blot analysis was performed as previously described (Dong et al., 2021). In brief, the hepatic tissue or cells were washed with ice-cold PBS and resuspended in ice-cold PBS containing a protease inhibitor cocktail (Beyotime, Shanghai, China) for 30 min. After centrifugation at 12,000×g for 10 min, the supernatants were collected and the protein concentrations were tested using a BCA protein assay kit (Beyotime, Shanghai, China). The supernatants were added with a sodium dodecyl sulfate (SDS) loading buffer to achieve a concentration of 20 mg/mL and the mixture was boiled at 95°C for 10 min. Equal amounts of protein samples were subjected to SDS-PAGE and then the resultant bands were transferred onto a polyvinylidene fluoride membrane (Millipore, Billerica, United States). The membranes were blocked with 5% non-fat milk at room temperature for 1 h and then incubated with various primary antibodies overnight at 4°C, followed by incubation with the appropriate horseradish peroxidase-conjugated secondary antibody for 1 h at room temperature. The enhanced chemiluminescence (Meilunbio, Dalian, China) was used to detect protein bands. The ChemiDoc MP Imaging System (Bio-Rad, California, United States) was used to capture images of the bands. GAPDH served as an internal control.

### Cell culture

The LO2 cell line was purchased from Wuhan Procell Life Science & Technology Co., Ltd. and cultured in complete medium containing 10% fetal bovine serum (FBS), 1% liquid medium supplement, and 1% penicillin/streptomycin. All cells were cultured in an incubator at 37°C with 5% carbon dioxide (CO2) and 95% air.

### Cell viability assay

The cell viability was detected using the 3-(4,5-dimethylthiazol-2-yl)-2,5-diphenyltetrazolium bromide (MTT) assay after treatment with different doses of ponatinib. In brief, cells with a density of 1 × 10^3 were seeded into a 96-well plate and cultured overnight. Cells were treated with increasing doses of ponatinib for 24 h. After treatment, the cells were washed with PBS, added with MTT solution (0.5 mg/mL PBS) and incubated at 37°C for 4 h. After the MTT solution was removed, the cells were washed with PBS and DMSO was added to dissolve formazan. The absorbance was analyzed at a wavelength of 490 nm using a spectrophotometer.

### Cell treatment

To gain insight into the effects of the MCD diet on hepatocytes, LO2 cells were cultured under methionine- and choline-free conditions (Song et al., 2023). Briefly, cells were initially cultured in DMEM-containing dishes and then transferred to 6-well plates for culture. When the cells reached approximately 80% confluence, the original DMEM was removed, and the cells were further cultured for an additional 24 or 48 h in DMEM without methionine and choline (MCD-mimicking medium, MCD). If drug treatment was required, the drugs were pre-dissolved in the MCD medium to achieve the desired concentration.

### Statistical analysis

Using the Statistical Product and Service Solutions (IBM SPSS) software, the Kolmogorov-Smirnov and Levene’s tests were conducted to assess the normal distribution and homogeneity of variances, respectively. *t*-test was performed on GraphPad Prism (GraphPad Software Inc., San Diego, CA) to compare data from two groups. One-way analysis of variance (ANOVA) was used for data from three or more groups data, followed by Bonferroni’s multiple comparisons test. Data were presented as mean ± SEM. *P* < 0.05 was considered statistically significant.

## Result

### Ponatinib reduced hepatic steatosis in MCD diet-fed mice

**FIGURE 1:**
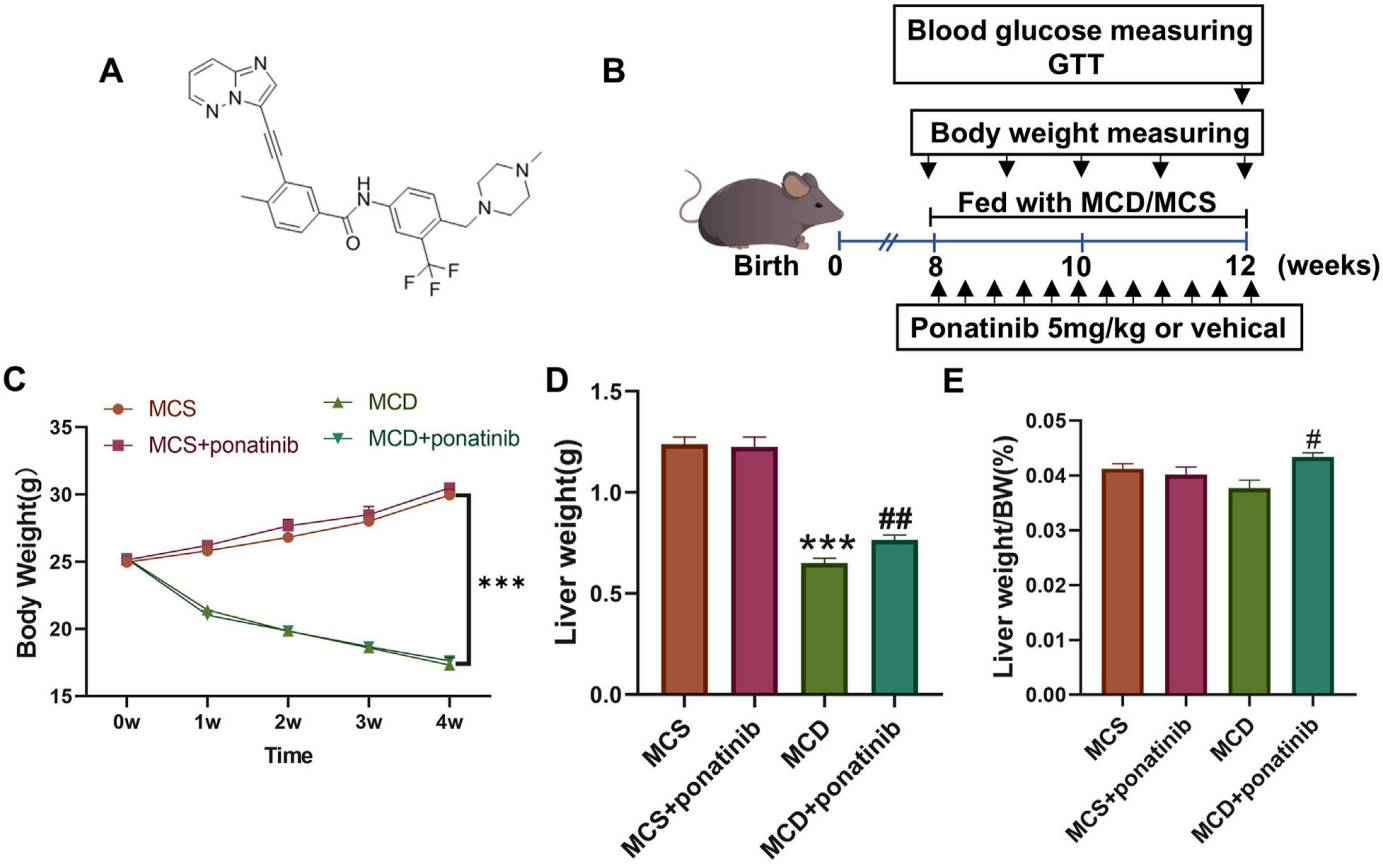
The effect of Ponatinib on body weight, liver weight, and liver index in mice. **(A)** Structural formula for ponatinib. **(B)** A Schematic diagram of the experimental protocol of the study ponatinib on the MCD model mice. **(C)** Body weight of each group of mice was measured weekly. **(D, E)** Liver weight **(D)** and liver weight/BW **(E)** were detected after 4 weeks of treatment in mice. n = 5. Data represent mean ± SEM. ^***^ *P* < 0.001 vs. MSD group. ^#^ *P* < 0.05, ^##^ *P* < 0.01 vs. MCD group.

**FIGURE 2:**
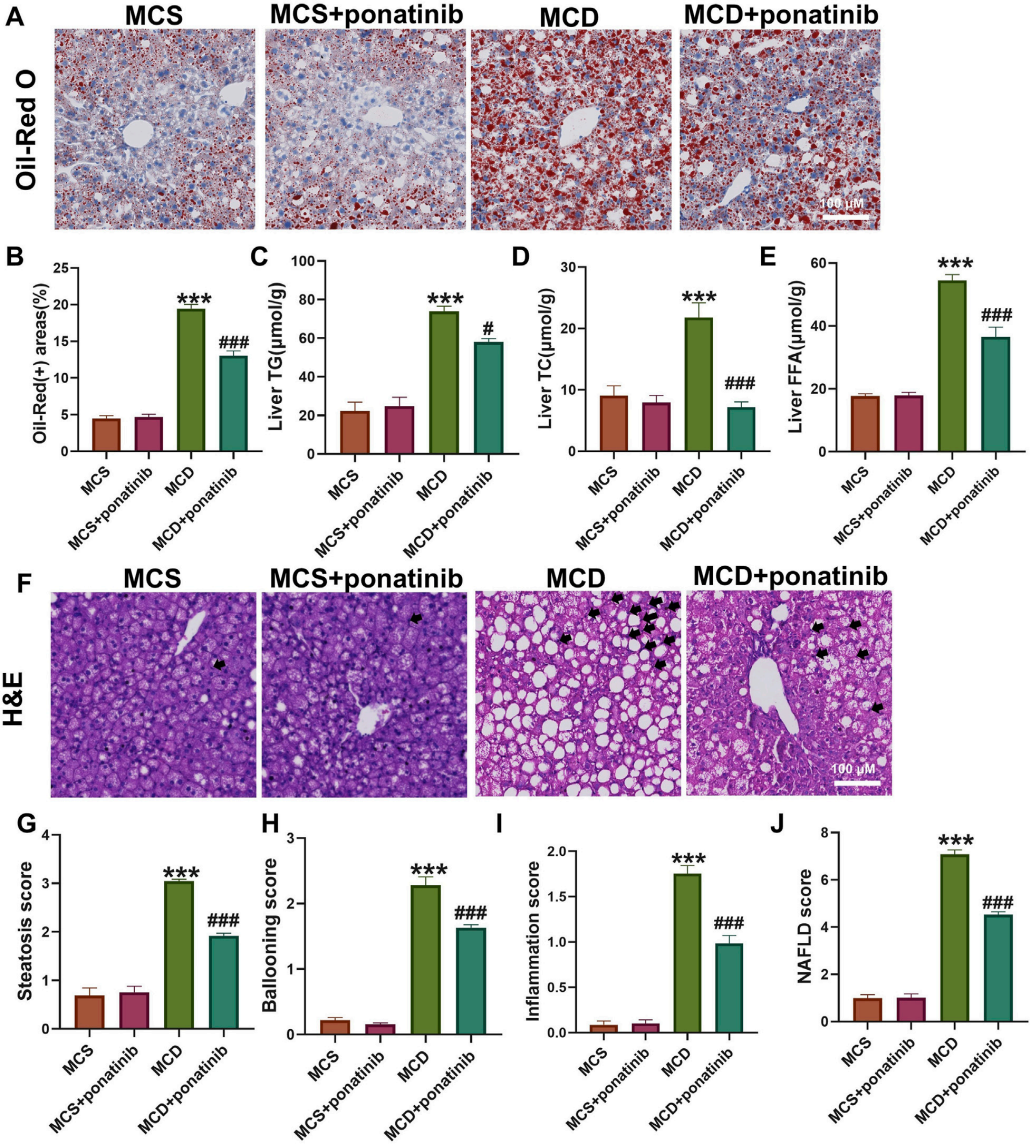
Ponatinib alleviated hepatic steatosis. **(A)** Representative Oil-Red O staining of mouse liver sections. **(B)** The positive area of Oil-Red O staining was calculated by image J. **(C–E)** Hepatic TG **(C)**, TC **(D)**, FFA levels **(E)** in mice with or without ponatinib treatment fed a MCD or MSD for 4 weeks. **(F)** Representative H&E staining of mouse liver sections. Black arrow denoted inflammation cells. **(G–J)** The NAFLD activity score (NAS) **(J)** was detected by histopathological analysis: steatosis **(G)**, ballooning **(H)**, and inflammation **(I)**. n = 5. Data represent mean ± SEM. ^***^ *P* < 0.001 vs. MSD group. ^#^ *P* < 0.05, ^###^ *P* < 0.001 vs. MCD group.

To elucidate the effect of ponatinib in hepatic steatosis, mice were fed with MCD diet for 4 weeks to established a NASH model and were orally administered with ponatinib (5 mg/kg) or vehicle for 4 weeks (Figure 1B). The results showed that the body weight and liver weight of the MCD group were lower compared to the MCS group (Figures 1C,D). We also observed that the liver/body mass index of mice in the MCD group was slightly lower compared to the MCS group; however, the difference did not reach statistical significance (Figure 1E). Additionally, there was no significant difference in body weight in the MCD group after ponatinib treatment while ponatinib treatment restored the liver weight and liver/body mass index of mice (Figures 1C–E). Further pathological analysis using Oil Red O staining revealed that hepatic fat accumulation was significantly reduced following ponatinib treatment in MCD diet mode (Figures 2A,B). Consistent with this, ponatinib remarkably attenuated the increase of hepatic triglycerides, cholesterol and FFA induced by MCD diet (Figures 2C–E). Steatosis and hepatocyte swelling were reduced after ponatinib treatment as observed by H&E staining (Figure 2F). Hepatic macrophages, which consist of Kupffer cells and recruited monocytes, constitute the largest innate immune cell population in the liver. In pathogenesis of NASH, Kupffer cells can drive homing of monocytes via chemokine signaling and contribute to the maintenance of heterogeneous macrophage populations (Sarkar et al., 2023). Our study demonstrated a reduction in the number of inflammatory cells subsequent to ponatinib treatment (Figure 2F). As assessed by the NAS system, ponatinib-treated mice exhibited a significantly lower NAFLD activity score compared to mice fed the MCD diet (Figures 2G–J).

### Ponatinib attenuated liver fibrosis and injury induced by MCD diet

**FIGURE 3:**
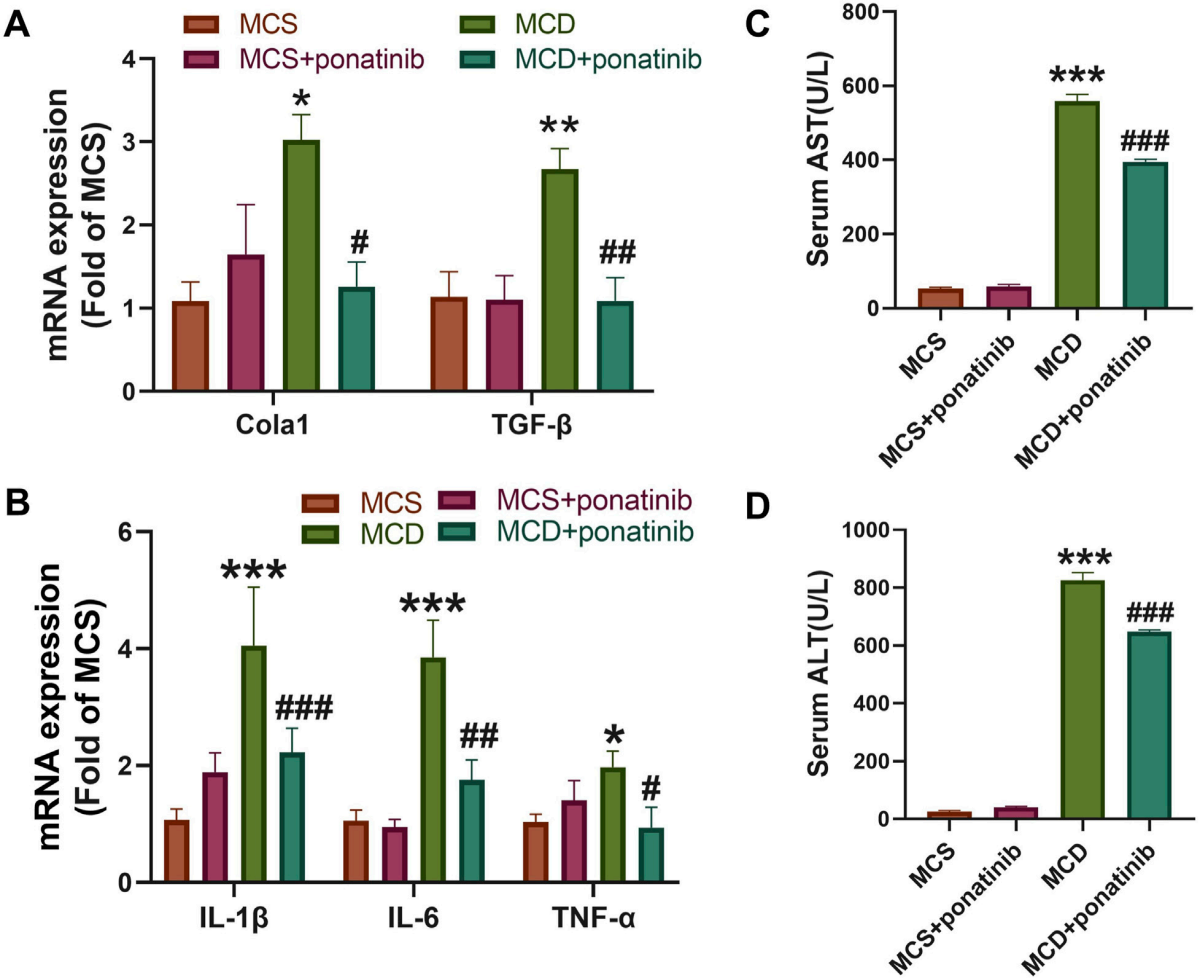
Ponatinib reduced the liver fibrosis and injury induced by MCD. **(A)** The relative mRNA levels of genes associated to fibrosis were tested by qPCR. **(B)** Hepatic gene expression associated with inflammation was measured by qPCR in mice fed with MCS or MCD diet and treated with ponatinib or vehicle for 4 weeks. **(C, D)** The levels of liver function markers AST **(C)** and ALT **(D)** in the serum of mice. n = 5, Data represent mean ± SEM. ^*^ *P* < 0.05, ^***^ *P* < 0.001 vs. MSD group. ^#^ *P* < 0.05, ^##^ *P* < 0.01, ^###^ *P* < 0.001vs. MCD group.

Hepatic steatosis is actually accompanied by fibrosis (Ren et al., 2024). The mRNA levels of genes related to fibrosis, such as the expression of Cola1 and TGF-β in liver tissue, were significantly decreased after ponatinib treatment compared with those in the MCD-diet group (Figure 3A). Furthermore, the increased expression of inflammatory factors, such as IL-1β, IL-6, TNF-α, were compromised after ponatinib treatment (Figure 3B). To further test whether ponatinib could alleviate hepatocyte injury, we detected the serum AST and ALT levels of mice. The results showed that the levels of ALT and AST in serum were significantly lower than those in MCD group after ponatinib treatment (Figures 3C,D).

### Ponatinib reduced serum lipid levels in MCD diet mice

**FIGURE 4:**
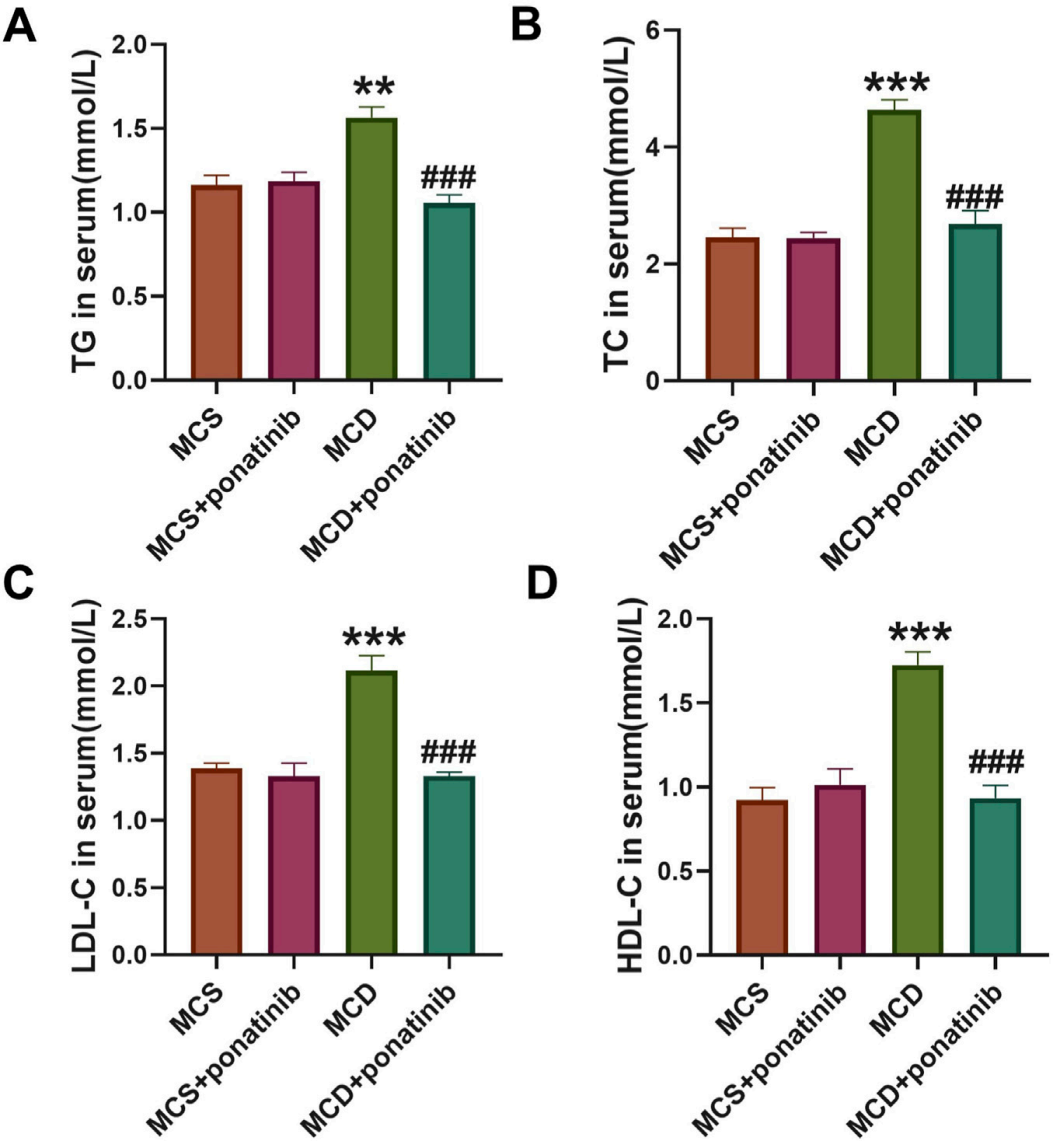
Ponatinib improved lipid levels in MCD diet-induced mice. **(A–D)** The levels of serum TG **(A)**, TC **(B)**, LDL-C **(C)**, HDL-C **(D)** were ascertained in mice fed either a MCD or MSD with or without ponatinib administration. n = 5, Data represent mean ± SEM. ^**^ *P* < 0.01, ^***^ *P* < 0.001 vs. MSD group. ^###^ *P* < 0.001vs. MCD group.

After 4-week feeding, the mice in MCD group exhibited higher levels of TC, TG, HDL-C, and LDL-C compared with those in MSD group (Figures 4A–D). Ponatinib intervention led to a significant reduction in the serum levels of TG, TC, HDL-C, and LDL-C among the mice fed with the MCD diet (Figures 4A–D).

### Ponatinib had no significant impact on the parameters of diabetes in MCD-fed mice

**FIGURE 5:**
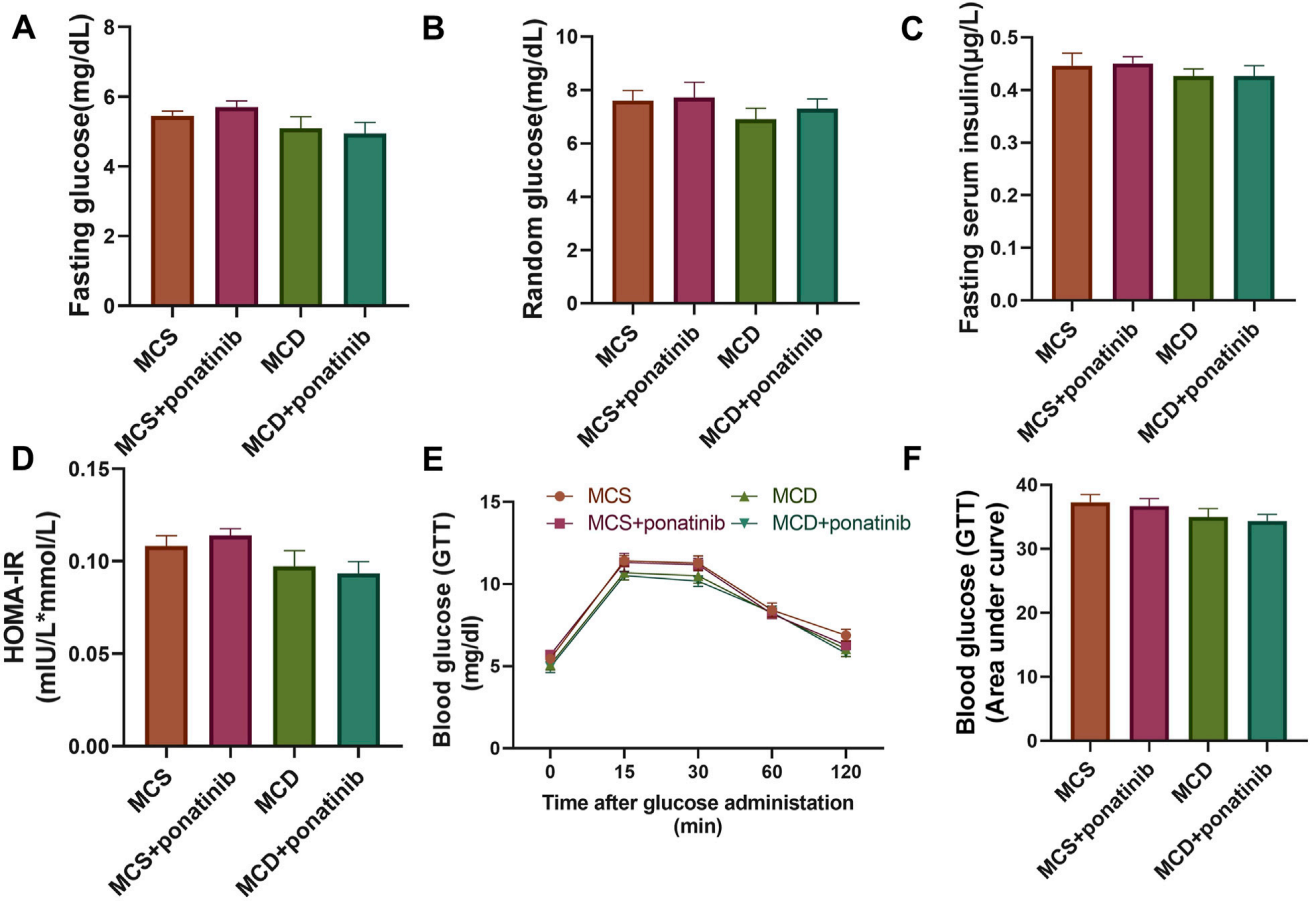
Ponatinib had no effect on blood glucose levels in MCD mice. **(A)** Fasting blood glucose levels were assessed in mice with ponatinib or vehicle fed either a MSD or MCD for 4 weeks. **(B)** The level of random blood glucose was measured in each group of mice fed either a MSD or MCD for 4 weeks. **(C)** Plasma insulin was measured in each group of mice. **(D)** HOMA-IR index was assessed by the levels of fasting blood glucose and fasting insulin in mice. **(E)** GTT was performed on mice after 4-week administration. **(F)** The area under the GTT curve was calculated by the Image J. n = 5. Data represent mean ± SEM.

Hepatic dysfunction is associated with glucose metabolism (Targher et al., 2021). To evaluate whether ponatinib affected glucose homeostasis in mice, the levels of random blood glucose, fasting blood glucose and fasting insulin were detected and HOMA-IR was calculated. The results suggested that there were no significant differences in random blood glucose, fasting blood glucose, fasting insulin and HOMA-IR between MCD group and MSD group after 4 weeks of feeding and ponatinib had no significant effect on blood glucose metabolism in mice fed with MCD or MSD diet (Figures 5A–D). The mice received an intraperitoneal injection of glucose to conduct a GTT, which indicated that glucose tolerance remained unaffected by either ponatinib or the MCD diet (Figures 5E,F). Therefore, these results suggested that ponatinib had no significant effect on glucose metabolism.

### Lipid deposition and inflammation induced by MCD-mimicking media were attenuated in human liver cell line-LO2 after ponatinib treatment

**FIGURE 6:**
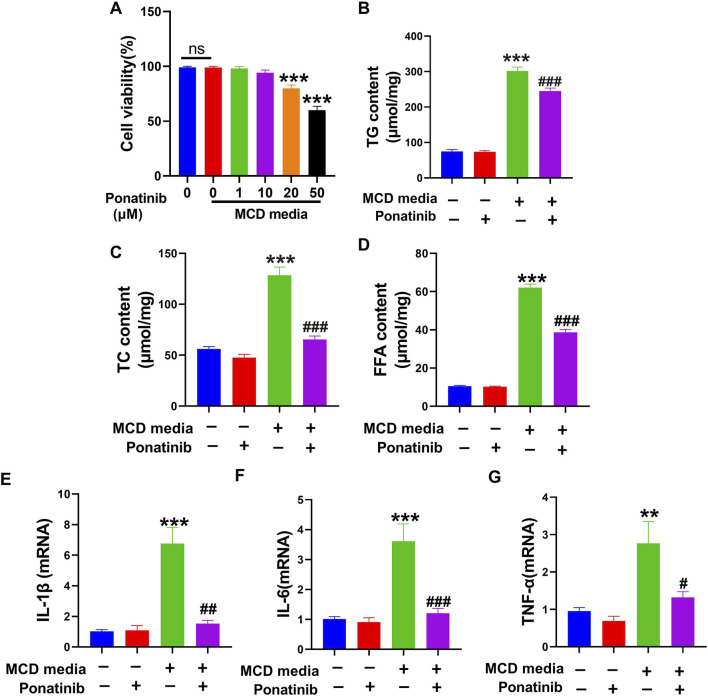
Ponatinib reduced lipid content and inflammation in L02 cells. **(A)** The effect of different doses of ponatinib on cell viability was examined by MTT assay. **(B)** Intracellular TG levels was detected in L02 cell with or without ponatinib treatment followed by MCD-mimicking media for 24 h. **(C)** Intracellular TC levels was obtained in LO2 cell with or without ponatinib treatment after MCD-mimicking media administration for 24 h. **(D)** Intracellular FFA levels was obtained in LO2 cell with or without ponatinib treatment after MCD-mimicking media administration for 24 h. **(E–G)** The relative mRNA levels of the proinflammatory markers were determined in LO2 treated with different conditions. n = 6. Data represent mean ± SEM.^**^ *P* < 0.01, ^***^ *P* < 0.001 vs. control group. ^#^ *P* < 0.05, ^##^ *P* < 0.01, ^###^ *P* < 0.001vs. model group.

Since ponatinib played a critical therapeutic role in MCD-induced NASH, we investigated whether NASH *in vitro* model was suppressed by ponatinib. The effects of ponatinib (0,1,10,20 and 50 μmol/L) on the viability of LO2 cells at 24 h were shown in Figure 6A. The viability of LO2 cells was not significantly affected by ponatinib treatment, except for treatment with 20 μmol/L or 50 μmol/L ponatinib for 24 h (Figure 6A). Therefore, LO2 cells were treated with a dose of 1 μmol/L ponatinib for 24 h in subsequent experiments. The content of TG, TC and FFA revealed that lipid deposition was lower in ponatinib-treated L02 cells than in vehicle-treated L02 cells cultured with MCD-mimicking media (Figures 6B–D). Moreover, the mRNA expression of inflammatory markers was inhibited in LO2 cells cultured with MCD-mimicking media after ponatinib treatment (Figures 6E–G).

### The damaged autophagy flow was repaired by ponatinib

**FIGURE 7:**
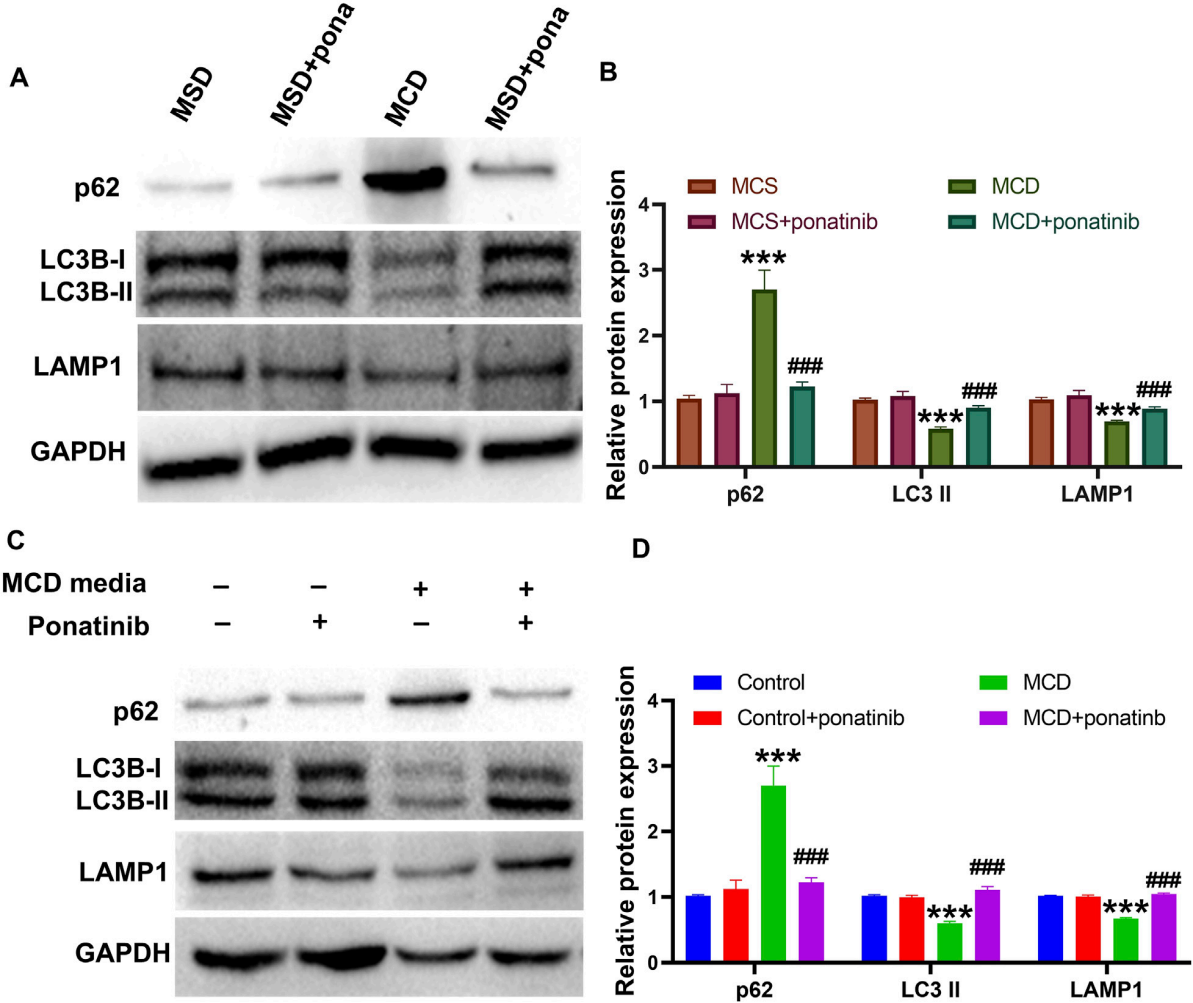
The autophagic flow damage caused by the MCD diet was relieved by the ponatinib. **(A–B)** The autophagy proteins LC3II, p62 and the lysosome protein LAMP1 were detected by immunoblotting in liver tissue of MCD-model mice treated with vehicle or ponatinib. The GAPDH protein was used to determine the relative protein levels after normalization. **(C–D)** The autophagy proteins LC3II, p62 and the lysosome protein LAMP1 were detected by immunoblotting in the LO2 cultured in MCD-mimicking media after vehicle or ponatinib treatment. Protein expression was normalized to GAPDH expression. n = 3. Data represent mean ± SEM. ^***^ *P* < 0.001 vs. control/MCD group. ^###^ *P* < 0.001vs. model group.

Previous studies have shown that autophagy is a critical metabolic pathway for lipid degradation and plays a key role in the treatment of NASH, indicated that pharmacological modulation of autophagy is a novel therapeutic approach for NASH (Scorletti and Carr, 2022). Therefore, we investigated whether ponatinib reduced lipid deposition and inflammation in hepatocytes by affecting autophagy. To investigate the role of ponatinib in autophagy, autophagy markers LC3B-I/LC3B II, lysosomal markers LAMP1 and autophagy substrate p62 were measured in MCD model mice. The results suggested that the protein levels of LC3B II and LAMP1 were decreased while the protein level of p62 was increased in MCD model mice, indicating the dysfunction of autophagic lysosomal degradation, while ponatinib could markedly reverse the protein expression of LC3B II, LAMP1 and p62, suggesting that the impaired lysosomal degradation function was restored after ponatinib treatment (Figures 7A,B). Similarly, the decreased protein expression of LC3B II and LAMP1 and the elevated protein level of p62 were inhibited by ponatinib in LO2 cells cultured with MCD-mimicking media (Figures 7C,D). These results suggested that ponatinib could repair the damage of autophagy in MCD model.

### The activity of TFEB was induced by ponatinib in MCD model

**FIGURE 8:**
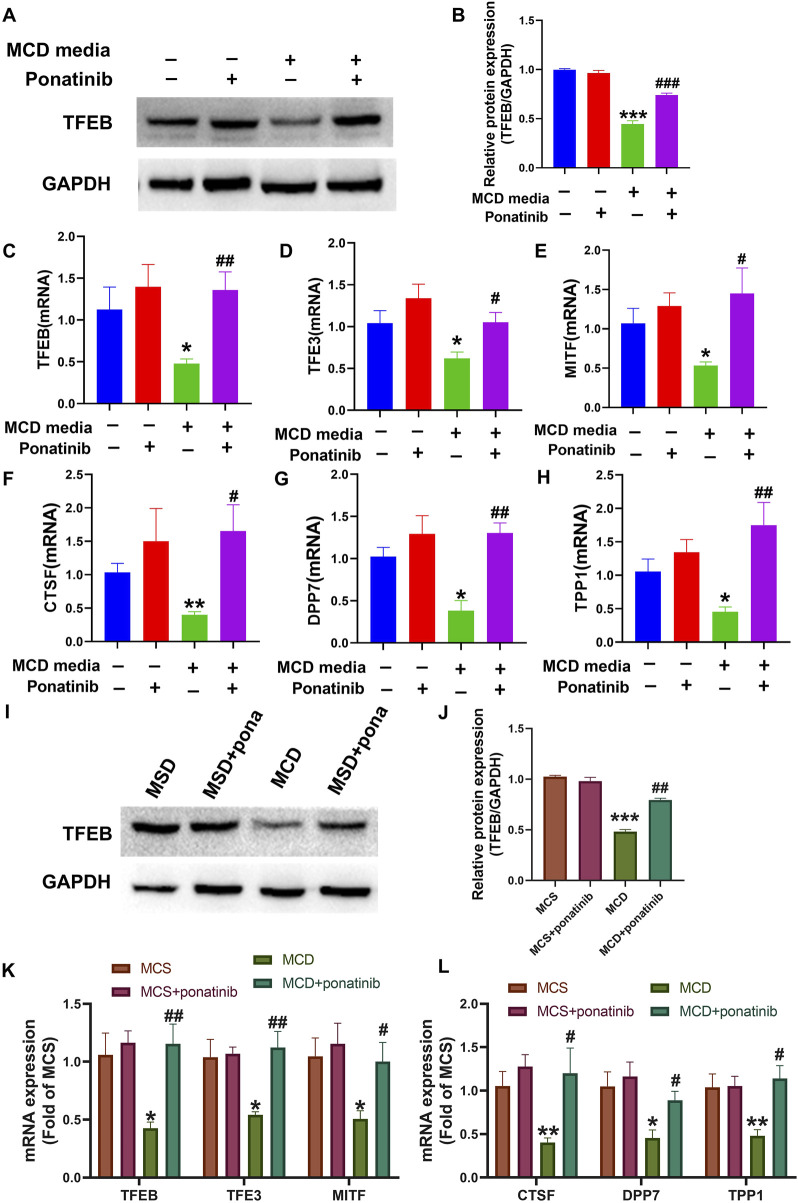
The transcription activity, mRNA and protein levels of TFEB were restored by the ponatinib. **(A–B)** The protein of TFEB was detected by immunoblotting in cells of MCD media treated with vehicle or ponatinib. The GAPDH protein was used to determine the relative protein level after normalization. n = 3. **(C–E)** The relative mRNA levels of MITF/TFE family were determined in LO2 cells treated with different conditions. n = 5. **(F–H)** The relative mRNA levels of TFEB target genes were determined in LO2 cells treated with different conditions. n = 5. **(I–J)** The protein of TFEB was detected by immunoblotting in liver tissue of MCD diet mice treated with vehicle or ponatinib. The GAPDH protein was used to determine the relative protein level after normalization. n = 3. **(K)** The relative mRNA levels of MITF/TFE family in liver tissue were tested by qPCR. n = 5. **(L)** The relative mRNA levels of TFEB target genes were determined in mice treated with different conditions. n = 5. Data represent mean ± SEM. ^*^ *P* < 0.05, ^**^ *P* < 0.01, ^***^ *P* < 0.001 vs. control/MCD group. ^#^ *P* < 0.05, ^##^ *P* < 0.01, ^###^ *P* < 0.001vs. model group.

Studies have shown that the increased lysosome production is closely related to the recovery of autophagy flow in NASH model, and TFEB plays an important role in the regulation of lysosome production (Zhang et al., 2018). The results showed that ponatinib could inhibit the decrease of TFEB level in MCD model, and ponatinib restored TFEB protein level in LO2 cells *in vitro* (Figures 8A,B). In addition, the mRNA level of the microphthalmia-associated transcription factor/transcription factor enhancer (MITF/TFE) family (TFEB, TFE3, MITF) and TFEB target genes, such as dipeptidyl-peptidase7 (DPP7), tripeptidyl peptidase I (TPP1), cathepsin F (CTSF) were restored treated with ponatinib *in vitro* (Figures 8C–H). Consistently, the results showed that ponatinib could inhibit the decrease of TFEB protein level in mice fed with MCD diet (Figures 8I,J). We further demonstrated that ponatinib restored the mRNA expression levels of the MITF/TFE family (TFEB, TFE3, and MITF) in mice fed with MCD diet (Figure 8K). Moreover, the decreased RNA levels of several known TFEB target genes, including DPP7, TPP1, and CTSF, were restored after ponatinib treatment (Figure 8L).

## Discussion

Epidemiological studies have demonstrated that NAFLD is the most prevalent chronic liver disease, affecting approximately 30% of the adult population. Among NAFLD patients, roughly 25% will progress to NASH, which is characterized by hepatocyte injury and inflammation. NASH can further advance to liver fibrosis, cirrhosis, and hepatocellular carcinoma, and has emerged as the second leading indication for liver transplantation (Adorini and Trauner, 2023). Therefore, it is a research focus to study the pathogenesis, prevention and treatment of NASH. This study revealed the beneficial effects of ponatinib on ameliorating hepatic inflammation and fibrosis by inducing autophagy. The study had two main findings. Firstly, ponatinib treatment alleviated hepatic steatosis, which was associated with enhanced autophagy. Secondly, the expression of TFEB was restored after ponatinib treatment, suggesting that ponatinib played a therapeutic role in NASH by upregulating TFEB, thereby facilitating the lysosomal clearance of autophagosomes.

Our study confirmed that ponatinib ameliorated MCD diet-induced NASH, including ameliorating steatosis, fibrosis and inflammation in the liver. Meanwhile, the results suggested that autophagy was inhibited in MCD-induced NASH while it was restored after ponatinib treatment. Autophagy is a crucial mechanism for maintaining cellular homeostasis, primarily through lysosomal-dependent pathways by eliminating damaged or dysfunctional organelles (Ren et al., 2024). Lipophagy is a selective autophagy that targets lipid droplets for degradation. It was first confirmed that autophagy is involved in the clearance of lipid droplets and triglycerides (Qian et al., 2021; Singh et al., 2009). The liver is an important site for fatty acid synthesis, metabolism and transportation (Xu et al., 2024). The most obvious pathological feature of NAFLD is the deposition of lipid droplets in hepatocytes, and the reduction of autophagy plays an important role in the progression of NAFLD (Ren et al., 2024; Singh et al., 2009). The accumulation of autophagy protein p62 was identified in the pathological sections of liver tissue in patients with severe fatty liver disease (Payan et al., 1986). Additionally, drugs with hepatic steatosis side effects have been proved to be related to the inhibition of hepatic autophagy (Stankov et al., 2012). For example, thymidine analogues of antiretrovirals, especially stavudine and Zidovudine, have been demonstrated to suppress autophagic activity. This phenomenon might potentially account for the elevated hepatic lipid content detected in individuals administered with these drugs (Begriche et al., 2006). These findings, in conjunction with our results, consistently supported the crucial role of autophagy in the modulation of liver fat levels within both animal models and human subjects. Moreover, it was indicated that aberrant autophagy significantly contributed to the pathogenesis of non-alcoholic fatty liver disease (Byrnes et al., 2022).

Studies have shown that impaired autophagy in NASH is attributed to abnormal lysosomal function (Steinberg and Hardie, 2023). Transcription factors of the MiT-TFE family, including TFEB, TFE3 and MITF, are capable of regulating lysosomal biogenesis, cellular energy homeostasis, and autophagy (Takla et al., 2023). Among them, TFEB, as a major regulator of autophagy, has the ability to regulate autophagy by promoting the production and function of lysosomes (Ren et al., 2024; Takla et al., 2023). In this study, we further found that ponatinib promoted lysosome production and restored autophagy in NASH model by antagonizing the downregulation of the transcription levels of MiT-TFE transcription factors including TFEB, TFE3 and MITF induced by MCD mimic medium and MCD diet.

Ponatinib, an oral active tyrosine kinase inhibitor, was used to intervene in drug-resistant mutations in BCR-ABL leukemia (Attwa et al., 2024). Owing to the multi-target characteristics of ponatinib, its administration in clinical practice has been associated with the increased incidence of arterial occlusion, which makes its clinical application need to be more cautious (Lin et al., 2022). Recent accumulating evidence suggested that ponatinib could play a therapeutic role in the intervention of non-cancer diseases such as pulmonary hypertension, pulmonary fibrosis, cerebral cavernous malformation, and aortic dissection (Kang et al., 2016; Qu et al., 2015; Choi et al., 2018; Zhang et al., 2023). Recent studies have demonstrated that ponatinib could improve dyslipidemia and atherosclerotic plaque formation in apolipoprotein E-deficient mice, suggesting that ponatinib exerted a significant role in lipid metabolism (Pouwer et al., 2018). In our prior study, it was demonstrated that ponatinib was capable of enhancing the metabolic profile of obese mice. However, owing to the limitation of ob/ob mice, which failed to progress to typical hepatitis or liver fibrosis, the impact of ponatinib on non-alcoholic hepatitis could not be further investigated (Lin et al., 2022). In this study, we used the methionine-choline-deficient model to further clarify the effect of ponatinib on NASH. Several recent studies have shown a potential relationship between ponatinib and autophagy. For example, enhanced autophagy could lessen the BCR-ABL-independent resistance of chronic myeloid leukemia cells to ponatinib (Kayabasi et al., 2017). Our research also indicated that ponatinib could improve NASH through autophagy.

Previous studies have consistently observed decreased expression of HDL-C in MCD models (Yu et al., 2019). Elevated levels of HDL-C are traditionally associated with anti-atherosclerotic and cardioprotective effects (von Eckardstein et al., 2023). However, recent research has revealed functional indicators of HDL, particularly cholesterol efflux capacity, HDL particle number, or different HDL proteins, are better predictors of atherosclerotic cardiovascular disease events than the level of HDL-C and elevated HDL-C levels achieved through drug therapy do not necessarily translate to a reduced risk of major adverse cardiovascular events (Investigators et al., 2011; Voight et al., 2012; von Eckardstein et al., 2023). Furthermore, our prior research demonstrated increased HDL-C expression in the NAFLD model of obese mice (Lin et al., 2022). Additionally, we observed MCD induced NASH model also exhibited elevated HDL-C levels in the model group. These findings may indicate the intricate nature of lipid and lipoprotein particles and their multifaceted role in the pathogenesis of metabolic diseases.

**FIGURE 9:**
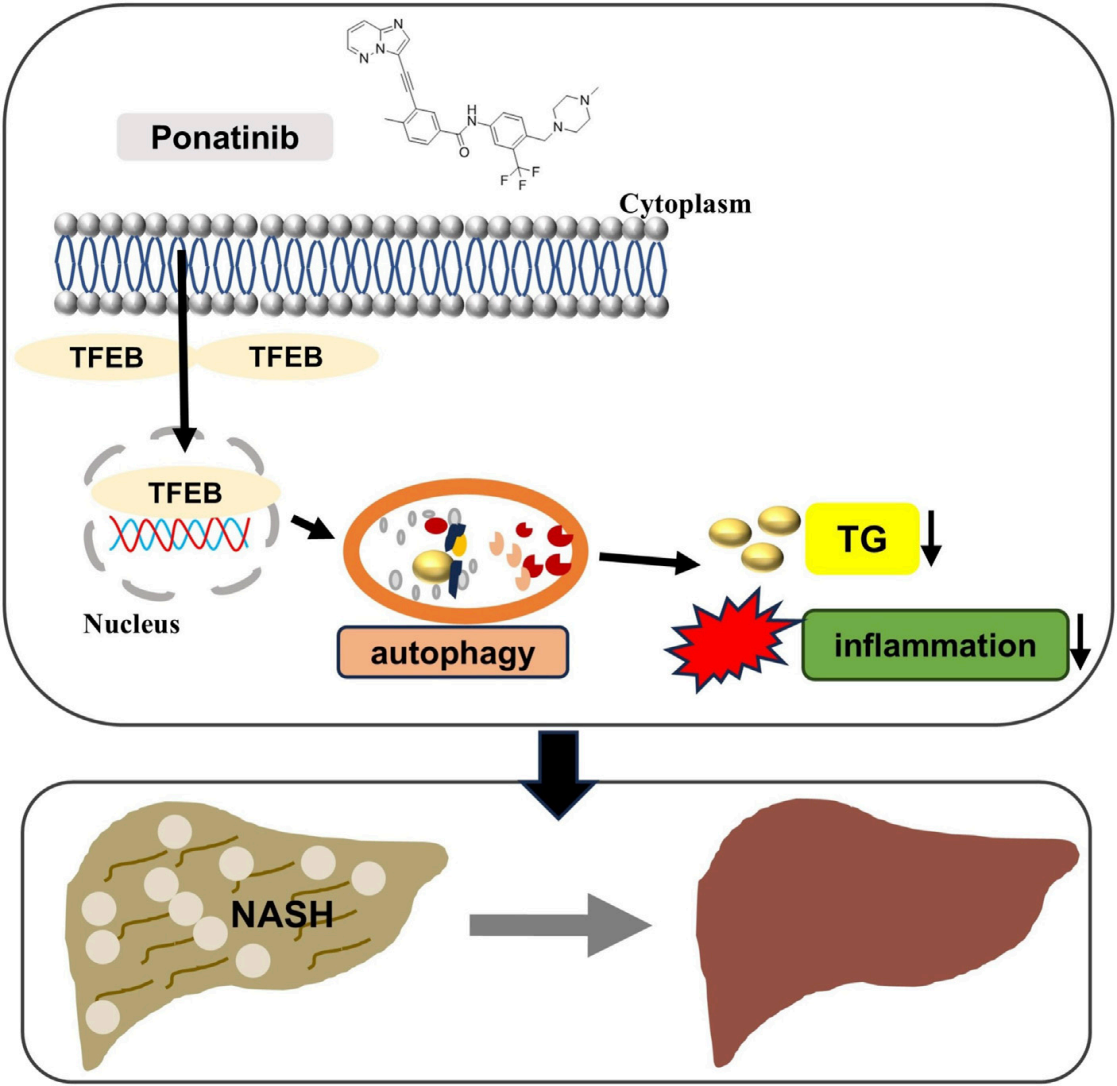
Schematic model of ponatinib improving hepatic steatosis via TFEB-autophagy in NASH mice. The protective effects of ponatinib on inhibiting excessive lipid accumulation in the liver and inhibiting inflammation depend on the TFEB-autophagy axis.

Recent evidence has shown that extrahepatic organs affect the progression of NAFLD, and progressive adipose tissue dysfunction and insulin resistance (IR) are key events in the development of NASH (Targher et al., 2021). Meanwhile, the progression of NAFLD is a major risk factor for type 2 diabetes (Hansen et al., 2023; Somm et al., 2021). Our results showed that there was no significant difference in blood glucose between the selected NASH model mice and normal mice, and ponatinib had no significant effect on blood glucose. The possible reason is the limitation of the MCD model, which differs from the metabolic characteristics of human NASH (Du et al., 2019). The mice do not exhibit overweight or obesity characteristics, and there is no IR with low serum insulin, low fasting blood glucose, and low leptin (Parlati et al., 2021; Rinella and Green, 2004). Ponatinib had previously been shown to improve insulin resistance in ob/ob mice, so this study could complement the limitations of our use of animal models (Lin et al., 2022). However, further study in a wider range of animal models is needed to extend the therapeutic effects of ponatinib to humans. Our study found that ponatinib could effectively ameliorate hepatic steatosis, inflammation and fibrosis in MCD-induced NASH mice by enhancing autophagy through increasing TFEB expression, and thus ponatinib may be a promising drug for the treatment of NASH (Figure 9, Graphical abstract).

## Data Availability

The original contributions presented in the study are included in the article/supplementary material, further inquiries can be directed to the corresponding author.
